# CD8^+^ T lymphocyte is a main source of interferon-gamma production in Takayasu’s arteritis

**DOI:** 10.1038/s41598-021-96632-w

**Published:** 2021-08-24

**Authors:** Yan-Long Ren, Tao-Tao Li, Wei Cui, Li-Min Zhao, Na Gao, Hua Liao, Jiang-Hui Zhang, Jun-Ming Zhu, Zhi-Yu Qiao, Shi-Chao Guo, Li-Li Pan

**Affiliations:** 1 Department of Cardiology, Beijing Lab for Cardiovascular Precision Medicine, Capital Medical University Affiliated Beijing Anzhen Hospital, Beijing, China; 2 Department of Rheumatology, Capital Medical University Affiliated Anzhen Hospital, 2 Anzhen Road, Chaoyang District, Beijing, China; 3 Lung and Vessel Disease, Beijing Institute of Heart, Beijing, China; 4 Department of Cardiovascular Surgery, Capital Medical University Affiliated Anzhen Hospital, Beijing, China

**Keywords:** Rheumatology, Immunology, Cytokines, Inflammation

## Abstract

Interferon-gamma (IFN-γ) is a cytokine involved in the pathogenesis of Takayasu’s arteritis (TAK). However, the source of IFN-γ in TAK patients is not fully clear. We aimed to investigate the source of IFN-γ in TAK. 60 TAK patients and 35 health controls were enrolled. The lymphocyte subsets of peripheral blood were detected by flow cytometry, cytokines were detected by Bio-plex. The correlation among lymphocyte subsets, cytokines and disease activity indexes was analyzed by person correlation. The level of serum IFN-γ in TAK patients was significantly increased (*P* < 0.05). The percentage of CD3^+^IFN-γ^+^ cells in peripheral blood CD3^+^ cells was significantly higher in TAK patients than that of healthy control group (*P* = 0.002). A higher proportion of CD3^+^CD8^+^IFN-γ^+^ cells/CD3^+^IFN-γ^+^ cells (40.23 ± 11.98% vs 35.12 ± 11.51%, *P* = 0.049), and a significantly lower CD3^+^CD4^+^IFN-γ^+^/ CD3^+^CD8^+^IFN-γ^+^ ratio (1.34 ± 0.62% vs 1.80 ± 1.33%, *P* = 0.027) were showed in the TAK group than that of control group. The CD3^+^CD8^+^IFN-γ^+^/CD3^+^IFN-γ^+^ ratio was positively correlated with CD3^+^IFN-γ^+^cells/ CD3^+^cells ratio (r = 0.430, *P* = 0.001), serum IFN-γ level (r = 0.318, *P* = 0.040) and IL-17 level (r = 0.326, *P* = 0.031). It was negatively correlated with CD3^+^CD4^+^IFN-γ^+^/CD3^+^IFN-γ^+^ ratio (r = − 0.845, *P* < 0.001). IFN-γ secreted by CD3^+^CD8 ^+^ T cells is an important source of serum IFN-γ in TAK patients.

## Introduction

Takayasu’s arteritis (TAK) is a vessel vasculitis commonly diagnosed by thickening and stenosis of arteries such as the aorta and its branches. The pathological signs of TAK are the chronic inflammatory lesions within the vessel wall without involvement of tissues outside the vessel wall ^1^. In the early stages there is a granulomatous inflammation and infiltration of lymphocytes and monocytes in the vascular wall. After the acute phase, adventitial fibrosis, and intimal smooth muscle cells proliferation, instead of the inflammatory process, mainly lead to stenoses, occlusions and aneurysms of the arterials ^2^.

Interferon-gamma (IFN-γ) is a kind of highly effective antiviral bioactive substance and a kind of inflammatory factor with extensive immunomodulatory effect^3^. The expression of INF-γ mRNA gene was higher in peripheral blood mononuclear cells and the tissue of aorta of TAK patients ^4,5^. Serum IFN-γ level can even reflect the activity of TAK disease ^6^. Moreover, the role of IFN-γ level is proved by the correlation with other biomarkers reflecting activity and proinflammatory cytokines in TAK^6^. It is known that IFN-γ is mainly released by activated CD4^+^T cells, macrophages, natural killer T cells and CD8^+^T cells ^7^. Experiments have demonstrated the important role for Th1 CD4^+^T cells in giant cell arteritis (GCA) ^8^ and TAK ^9^. However, the source of IFN-γ and the function of IFN-γ-producing CD8^+^T cells in TAK patients has not been fully clarified. The purpose of this study is to investigate the source of IFN-γ and the function of IFN-γ-producing CD8^+^T cells in TAK.

## Materials and methods

### Participants

The inclusion criteria of this study is mainly according to the criteria for classifying TAK developed by the American College of Rheumatology in 1990. The medical records of patients included were reviewed by two separated rheumatologists. Patients with other autoimmune diseases, liver or kidney dysfunction, cancer or infection were excluded. Baseline data including demographic data, symptoms, signs, laboratory tests and imaging material were collected. 35 healthy controls with well-balanced sex and age were enrolled. Method of detecting the cytokines and T lymphocyte subsets of peripheral blood were described in the following paragraph. All patient’s data were retrospectively collected from the Department of Rheumatology and Immunology, Beijing Anzhen Hospital, from December 2017 to February 2018.

### Flow cytometry

Cell phenotype was analyzed by flow cytometry. 1 ml heparinized blood samples were collected and stimulated with 20 ng/ml phorbol-12-myristate-13-acetate (PMA) and 1000 ng/ml ionomycin in the presence of Golgi-Stop (BD Biosciences) for 5 h, then incubated with anti-human mouse antibodies against CD3-FITC-A, CD4-BV510-A (BD Pharmingen), CD8-APC-H7 (BD Pharmingen), IFN-γ-PE-B27(BD Pharmingen). In all experiments, a control antibody of the respective IgG isotype was included. Flow assay was detected using FACS Calibur flow cytometer (BD, USA). Data was analyzed with FlowJo v.7.6.4 software (Tree Star).

### Cytokine assay

Serums of TAK patients and healthy controls were collected. The serum cytokine levels of IFN-γ, IL-17 and TNF-α were detected using Bio-Plex Pro Human Cytokine 27-plex Assay, conducted according to the manufacturer’s instructions.

### Disease activity assessment

The Indian Takayasu Clinical Activity Score (ITAS) and ITAS-A were used to evaluate the disease activity. ITAS forms were filled cross-sectionally for baseline for all clinical features. When the scoring system combined either C-reactive protein (CRP) or erythrocyte sedimentation rate (ESR), acute-phase reactants were more accurately identified.

### Pathological staining of aortic tissue

Human aortic specimens were fixed in 4% neutral formalin for 24 h, embedded in paraffin, sectioned. Aortic Sects. (4 μm) were stained with primary antibodies for CD8 (Abcam, 1:200 dilution), INF-γ (Abcam, 1:200 dilution) at 4 °C overnight, then with secondary antibodies at room temperature for 0.5–1 h and detected with 3,3′-diaminobenzidine. The ratio of the specific positive area in the aortic tissue area was calculated (area fraction, %).

For immunofluorescence cryosections were stained with anti- CD8 (1:100) and anti- INF-γ (1:100), and then incubated with FITC and tetramethylrhodamine isothiocyanate-conjugated secondary antibodies.

### Statistical analysis

The baseline characteristics of patients were presented as mean ± SD for continuous variables and compared using the t test if the data were of normal distribution. Non-normally distributed data demonstrated as medians (P25, P75) were compared with Wilcoxon signed-rank test. The correlation among lymphocyte subsets, cytokines and disease activity indexes was analyzed by person correlation. All analyses were carried out using the SPSS statistical software V. 20.0. Furthermore, we added conditional formatting to Excel (Microsoft® Excel® 2019) cells to display color heatmap of all cytokines.

### Ethics approval

The study was conducted in accordance with the ethical principles of the Declaration of Helsinki and approved by the Clinical Research Ethics Board of Beijing Anzhen Hospital, Capital Medical University (approval number: 2019054X).

### Consent to participate

Informed consent was obtained from all individual participants included in the study.

## Results

### Clinical manifestations of TAK patients

Among 60 TAK patients, 56 were female which accounting for 93.3%. The average age of TAK patients was 38.50 years old, the median course of disease was 48 months, the median of ESR, CRP, Kerr score, ITAS 2010 and ITAS-A was 10 mm/1 h, 3.12 mg/l, 2, 1.0 and 3.5 respectively. The most common Numano type was Type V which accounting for 51.7%, followed by Type II B (20.0%). Glucocorticoid (81.0%) was the most used, followed by methotrexate (MTX 57.1%), mycophenolate mofetil (MMF, 33.3%), IL-6 receptor antagonist (IL-6RI, 33.3%) and cyclophosphamide (CTX, 28.8%) (Table 1).

**Table 1:** Clinical characteristic of TAK patients.

Parameters	TAK (n = 60)
Age, years	38.50 ± 11.70
Female, n (%)	56 (93.3)
Disease duration, months	48.0 (12.0, 120.0)
ESR (mm/1 h)	10 (5.0, 14.0)
CRP (mg/l)	3.12 (0.63, 6.60)
Kerr score	2.0 (0.0, 2.0)
ITAS2010	1.0 (0.0, 6.00)
ITAS-A	3.5 (0.00, 7.75)
**Numano type, n (%)**	
I	9 (15.0)
IIa	1 (1.7)
IIb	12 (20.0)
III	5 (8.3)
IV	2 (3.3)
V	31 (51.7)
New diagnosed, n (%)	39 (65.0)
**Treatment, n (%)**	21 (35.0)
GC	17/21 (81.0)
CTX	6/21 (28.6)
MMF	7/21 (33.3)
MTX	12/21 (57.1)
HCQ	3/21 (5.0)
IL-6RI	7/21 (33.3)

*ESR* erythrocyte sedimentation rate, *CRP* C-reactive protein, *GC* glucocorticoid, *CTX* cyclophosphamide, *MTX* methotrexate, *MMF* mycophenolate mofetil, *HCQ* hydroxychloroquine.

### Comparison of lymphocyte subsets and cytokines between TAK patients and control group

The peripheral blood lymphocyte subsets of 35 age and sex matched healthy controls were compared with those of TAK patients. Our results showed there was no difference in the percentage of CD3^+^ lymphocytes between TAK patients and healthy control (TAK = 53.69 ± 17.99% vs Control = 50.98 ± 14.68%, *P* = 0.462). The percentages of CD4^+^ lymphocytes and CD8^+^ lymphocytes in peripheral blood from TAK patients and control group were not significantly different: 63.28 ± 8.34% vs. 63.99 ± 8.02%, *P* = 0.692 and 32.76 ± 7.52% vs. 30.35 ± 8.11%, *P* = 0.155, respectively. Meanwhile, the ratio of CD4^+^ /CD8^+^ lymphocytes between two groups was similar (TAK = 2.08 ± 0.70% vs Control = 2.33 ± 0.91%, *P* = 0.147) (Table 2).

**Table 2:** Comparison of lymphocyte subsets between TAK patients and control group.

Parameters	TA (n = 60)	Control (n = 33)	*P* value
Age, years	38.50 ± 11.70	36.12 ± 6.67	0.286
Female, n, (%)	56(93.3)	28(84.8)	0.185
CD3^+^/lymphocytes, (%)	53.69 ± 17.99	50.98 ± 14.68	0.462
CD4^+^/lymphocytes, (%)	63.28 ± 8.34	63.99 ± 8.02	0.692
CD8^+^/lymphocytes, (%)	32.76 ± 7.52	30.35 ± 8.11	0.155
CD4^+^/CD8^+^, (%)	2.08 ± 0.70	2.33 ± 0.91	0.147

**Figure 1:**
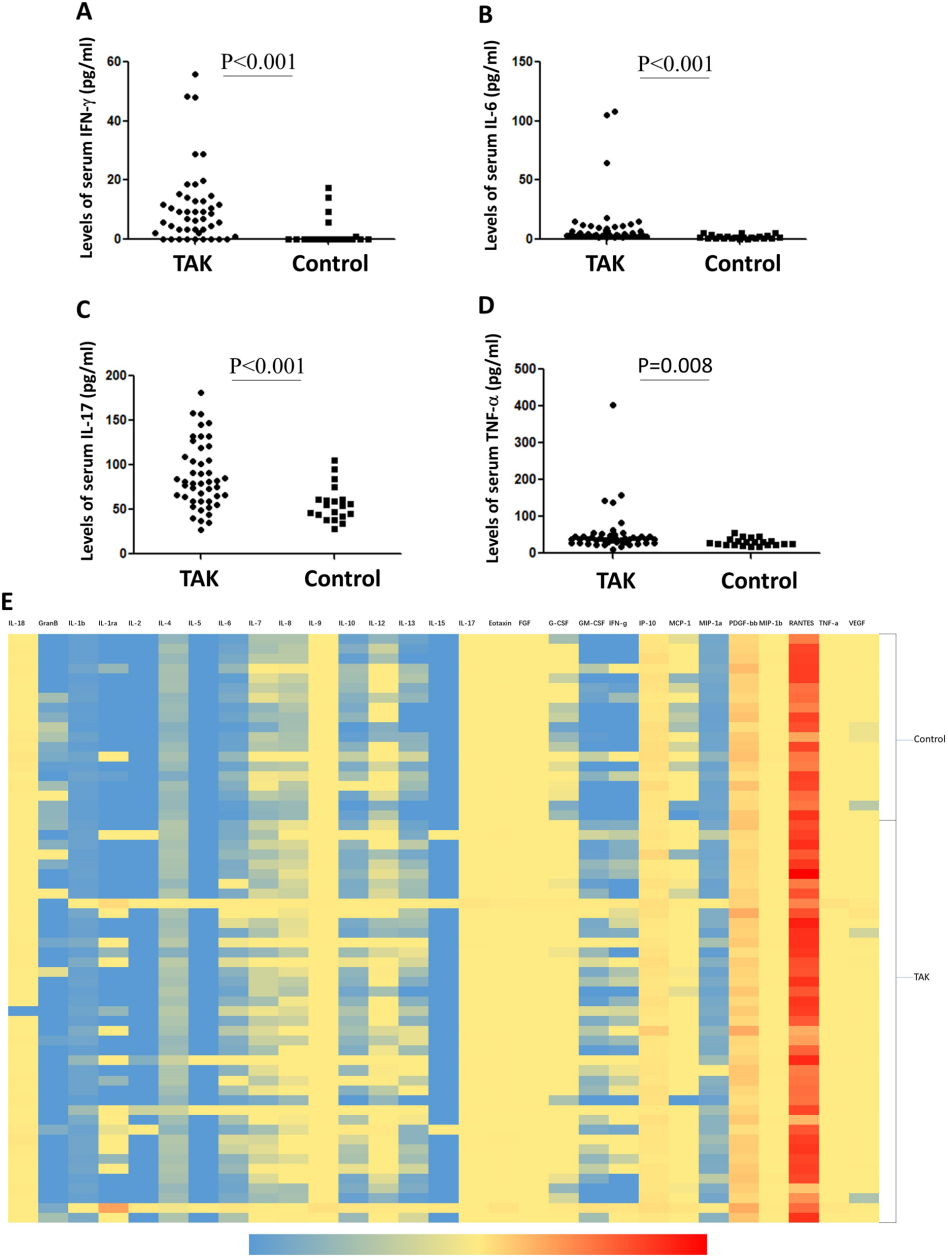
Serum cytokine levels in patients with 42 TA patients and 18 healthy controls. (**A**) The level of serum IFN-γ in TA patients was significantly higher than that in healthy controls (*P* < 0.001); (**B**) the level of serum IL-6 was significantly higher than that in the control group (*P* < 0.001); (**C**) the level of serum IL-17 in Ta Group was significantly higher than that in the control group (*P* < 0.001); (**D**) the level of serum TNF-α in Ta Group was significantly higher than that in the control group (*P* = 0.008); (**E**) the heatmap of serum cytokines in peripheral blood of 42 patients with TAK and 18 healthy controls.

We measured the levels of cytokines in peripheral blood of 42 patients with TAK and 18 healthy controls. IFN-γ levels were significantly elevated in patients with TAK relative to healthy controls [6.79 (0.23, 12.66) pg/ml vs. 0.00 (0.00, 0.23) pg/ml, *P* < 0.001]. The level of serum IL-6 was 4.13 (2.95, 9.93) pg/ml in TAK patients, which was significantly higher than that in control group [1.47 (0.81, 2.67) pg/ml, (*P* < 0.001)]. The level of serum IL-17 and TNF-α in TAK patients was significantly higher than control group [79.32 (59.31106.09) pg/ml vs. 55.62 (43.53, 62.05) pg/ml (*P* < 0.001), 38.59 (27.78, 46.97) pg/ml vs 26.83 (22.80, 34.60) pg/ml (*P* = 0.008) respectively] (Fig. 1).

### Comparing the proportion of CD3^+^CD8^+^IFN-γ^+^cells in CD3^+^IFN-γ^+^ cells between the TAK group and the control group

**Figure 2:**
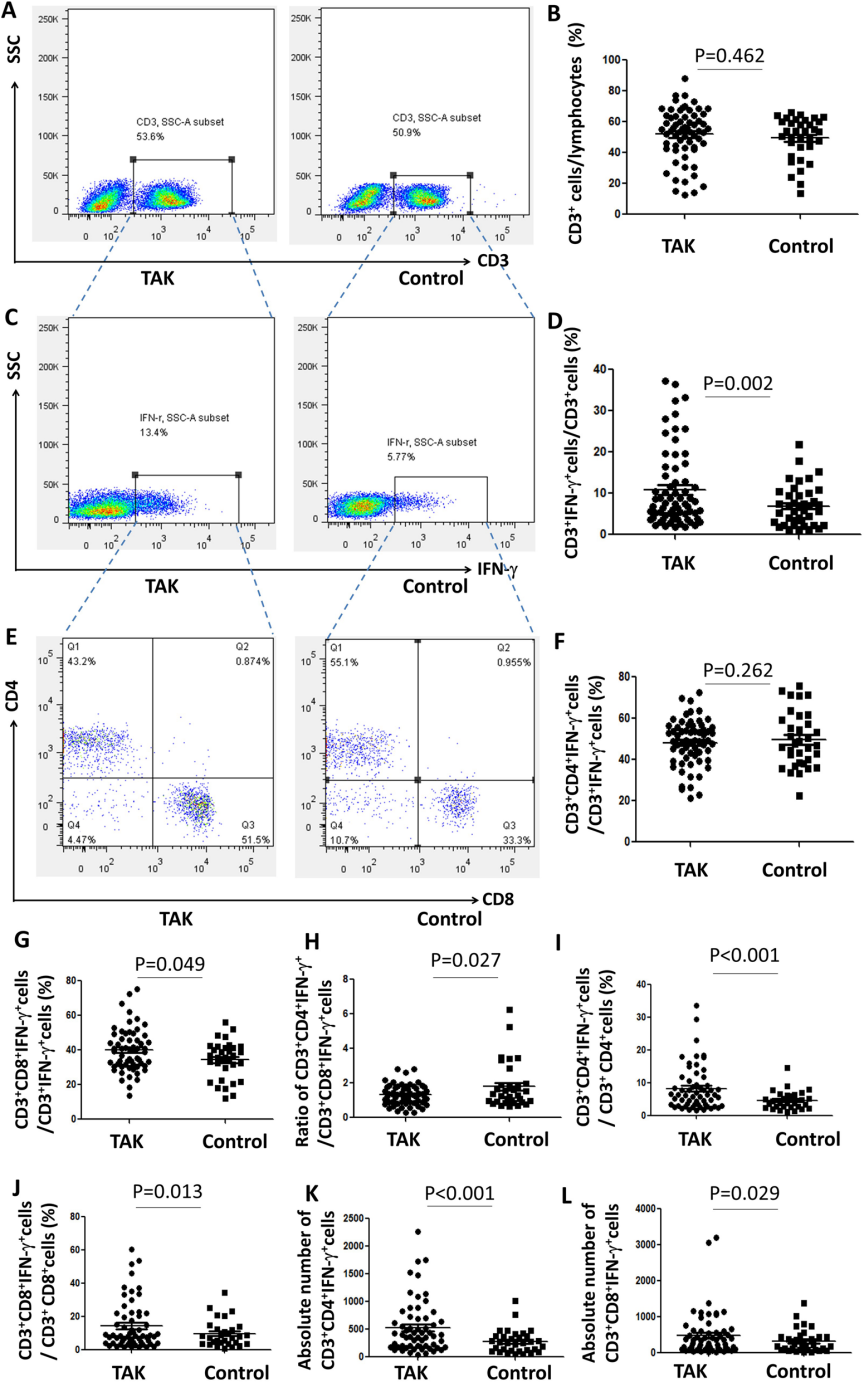
Comparing the proportions of CD3^+^CD8^+^IFN-γ^+^cells in CD3^+^IFN-γ^+^cells between the TAK group (n = 60) and the control group (n = 35). (**A,B**) The proportion of CD3^+^ cells in lymphocytes in TAK group and control group (*P* = 0.462). (**C,D**) The proportion of CD3^+^IFN-γ^+^ cells in CD3^+^ cells in TAK group and control group (*P* = 0.002). (**E–G**) The proportion of CD3^+^CD4^+^IFN-γ^+^cells (*P* = 0.262) and CD3^+^CD8^+^IFN-γ^+^cells (*P* = 0.049) in CD3^+^IFN-γ^+^ cells between two groups. (**H**) The ratio of CD3^+^CD4^+^IFN-γ^+^/ CD3^+^CD8^+^IFN-γ^+^ in the TAK group and control group (*P* = 0.027). (**I**) The CD3^+^CD4^+^IFN-γ^+^cells in CD3^+^ CD4^+^cells between two groups (*P* < 0.001). (**J**) The CD3^+^CD8^+^IFN-γ^+^cells in CD3^+^ CD8^+^cells between two groups (*P* = 0.013). (**K**) The absolute number of CD3^+^CD4^+^IFN-γ^+^cells between two groups (*P* < 0.001). (**L**) The absolute number of CD3^+^CD8^+^IFN-γ^+^cells between two groups (*P* = 0.029).

We compared MFI of IFN-γ^+^ in CD4^+^ and CD8^+^ T lymphocyte between TAK patients and control group, there was no significantly deference between the two group [(1467.40 ± 454.49 vs1436.25 ± 416.14, *P* = 0.713), ( 5950.44 ± 1785.25 vs 5980.80 ± 1418.53, *P* = 0.925) respectively] (Table 3). The proportion of CD3^+^CD4^+^IFN-γ^+^cells in CD3^+^ CD4^+^cells and CD3^+^CD8^+^IFN-γ^+^cells in CD3^+^ CD8^+^cells was significantly higher in TAK group respectively[8.16 ± 6.76% vs 4.56 ± 2.84%, *P* < 0.001, 14.29 ± 14.34% vs 9.59 ± 8.01%, *P* = 0.013]. The absolute number of CD3^+^CD4^+^IFN-γ^+^cells and CD3^+^CD8^+^IFN-γ^+^cells was significantly higher in TAK group respectively[523.1 ± 473.5 vs 273.3 ± 208.8, *P* < 0.001, 298.0 (97.8, 598.5) vs 213.0 (76.5, 430.5), *P* = 0.029]. To further explore the source of IFN-γ, we examined the proportion of IFN-γ^+^ cells in CD3^+^ cells. The proportion of IFN-γ^+^ cells in CD3^+^ cells was significantly higher in TAK group (11.14 ± 9.67% vs 5.61 ± 3.86%, *P* = 0.002). When comparing the proportion of CD3^+^CD4^+^IFN-γ^+^cells and CD3^+^CD8^+^IFN-γ^+^cells in CD3^+^IFN-γ^+^ cells, the higher proportion of CD3^+^CD8^+^IFN-γ^+^cells (40.23 ± 11.98% vs 35.12 ± 11.51%, *P* = 0.049), the similar proportion of CD3^+^CD4^+^IFN-γ^+^cells (47.39 ± 10.73% vs 50.23 ± 13.03%, *P* = 0.262) and a significantly lower CD3^+^CD4^+^IFN-γ^+^/ CD3^+^CD8^+^IFN-γ^+^ ratio (1.34 ± 0.62% vs 1.80 ± 1.33%, *P* = 0.027) were showed in the TAK group (Fig. 2).

**Table 3:** Comparison MFI of IFN-γ^+^ in T lymphocyte between TAK patients and control group.

Parameters	TA (n = 60)	Control (n = 33)	*P* value
MFI of IFN-γ^+^ in CD4^+^ T lymphocyte	1467.40 ± 454.49	1436.25 ± 416.14	0.713
MFI of IFN-γ^+^ in CD8^+^ T lymphocyte	5950.44 ± 1785.25	5980.80 ± 1418.53	0.925

### The relationship of CD3^+^CD8^+^IFN-γ^+^/CD3^+^IFN-γ^+^ ratio with parameters of disease activity

**Figure 3:**
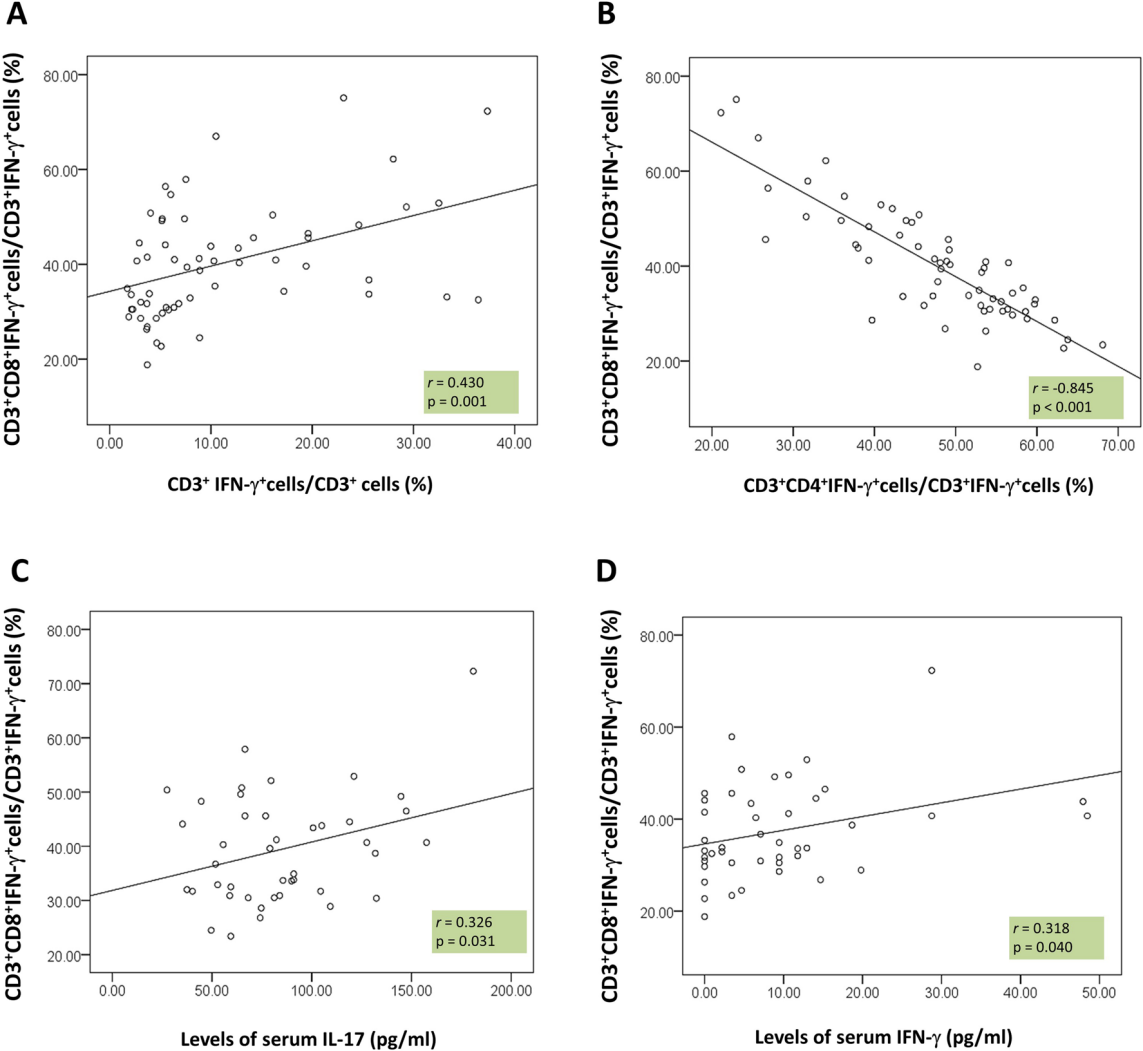
The relationship of CD3^+^CD8^+^IFN-γ^+^/CD3^+^IFN-γ^+^ ratio with other parameters. The CD3^+^CD8^+^IFN-γ^+^/CD3^+^IFN-γ^+^ ratio was positively correlated with (**A**) CD3^+^IFN-γ^+^cells/ CD3^+^cells ratio (r = 0.430, *P* = 0.001), (**C**) IL-17 level (r = 0.326, *P* = 0.031) and (**D**) serum IFN-γ level (r = 0.318, *P* = 0.040). The negative correlation between CD3^+^CD8^+^IFN-γ^+^/CD3^+^IFN-γ^+^ ratio and (**B**) CD3^+^CD4^+^IFN-γ^+^/CD3^+^IFN-γ^+^ ratio was found (r = − 0.845, *P* < 0.000).

The CD3^+^CD8^+^IFN-γ^+^/CD3^+^IFN-γ^+^ ratio was positively correlated with CD3^+^IFN-γ^+^cells/ CD3^+^cells ratio (r = 0.430, *P* = 0.001), serum IFN-γ level (r = 0.318, *P* = 0.040) and IL-17 level (r = 0.326, *P* = 0.031). Meanwhile, the negative correlation between CD3^+^CD8^+^IFN-γ^+^/CD3^+^IFN-γ^+^ ratio and CD3^+^CD4^+^IFN-γ^+^/CD3^+^IFN-γ^+^ ratio was found (r = − 0.845, *P* < 0.001) (Fig. 3). The parameters of disease activity, including ESR (r = 0.092, *P* = 0.483), CRP (r = 0.051, *P* = 0.700), NIH (r = 0.046, *P* = 0.738), ITAS2010 (r = 0.161, *P* = 0.237) and ITAS-A (r = 0.188, *P* = 0.166), were not associated with the CD3^+^CD8^+^IFN-γ^+^/CD3^+^IFN-γ^+^ ratio (Supplementary Fig. 1).

### CD8 and IFN-γ expressions in the aortic wall of TAK patients

**Figure 4:**
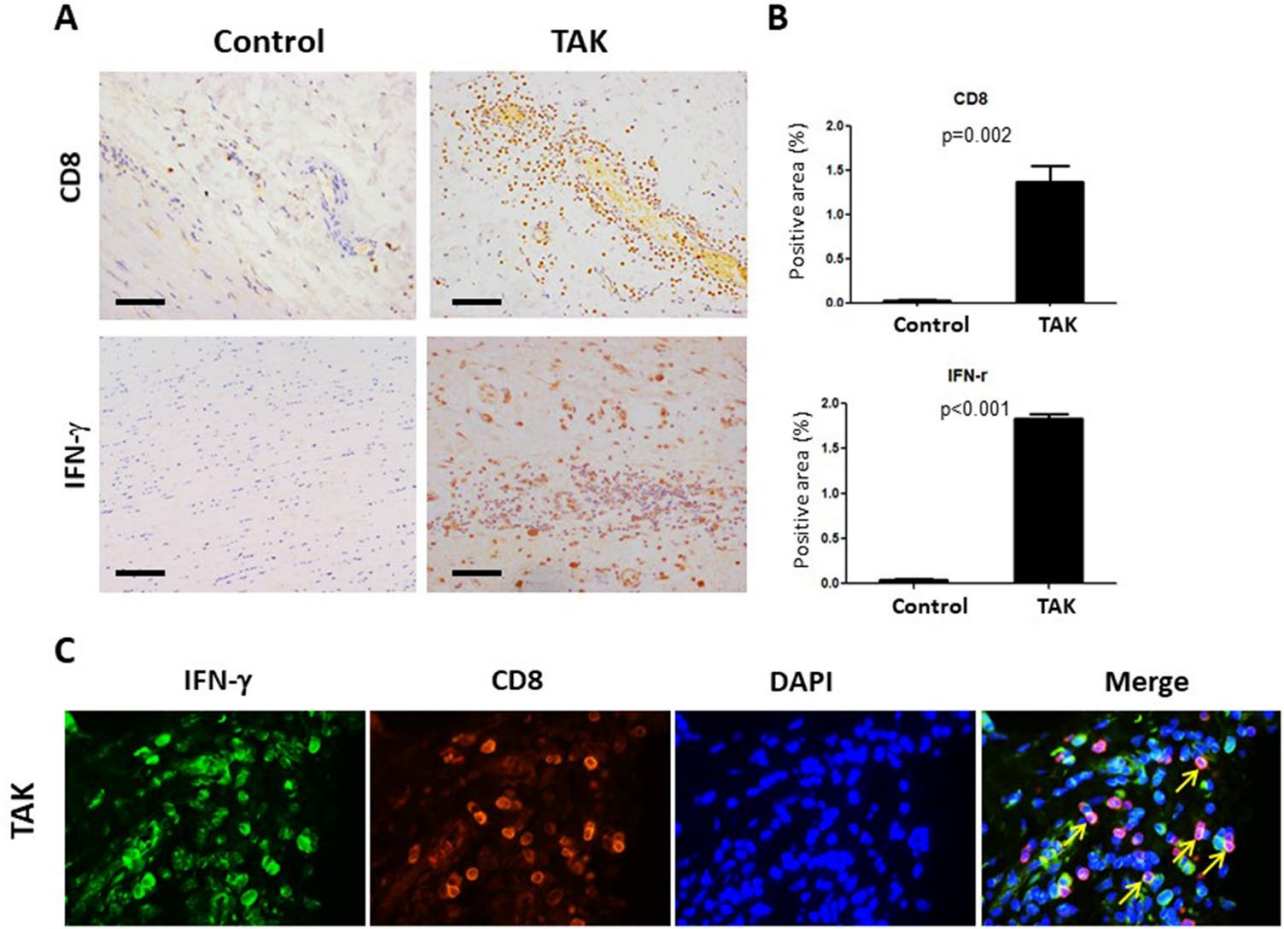
CD8 and IFN-γ expressions in the aortic wall of TAK patients. (**A**) CD8 and IFN-γ staining in the aortic wall of TAK patients and control group. (scale bars, 100 μm; n = 3 in each group). (**B**) The quantification of CD8 and IFN-γ staining in the two groups (n = 3 in each group). (**C**) Immunofluorescence staining stained for CD8 (green) and IFN-γ (red) in the aortic wall of TAK patients. Nuclei were shown in blue with 4′,6-diamidino-2-phenylindole (DAPI) staining.

Besides, we detected the expressions of CD8 and IFN-γ in the aortic wall of TAK patients and atherosclerosis patients. Immunohistochemical staining showed the increased infiltration of CD8^+^ cells (1.36% ± 0.26% vs. 0.03% ± 0.01%, *P* = 0.002) and IFN-γ^+^ cells (1.83% ± 0.09% vs. 0.04% ± 0.01%, *P* < 0.001) in the aortic tissue of TAK patients, compared with the atherosclerosis patients (A-B). Immunofluorescence staining indicated immunofluorescence staining showed that CD8^+^cells could produce IFN-γ (C) (Fig. 4).

## Discussion

Previous studies have indicated that IFN-γ was associated with pathogenic events in the inflammatory reaction of vessel wall. It play important role in activating the endothelial cells, stromal cells, dendritic cells and macrophages, inducing vascular endothelial growth factor (VEGF) and promoting neo-angiogenesis^10^. In our search, we found that the serum IFN-γ of TAK patients was significantly higher than that of healthy control people. To further explore the source of IFN-γ in TAK, we detected the proportion of IFN-γ^+^cells in the peripheral blood CD3^+^ cells of TAK patients. The results showed that the proportion of CD3^+^IFN-γ^+^ cells in the CD3^+^cells of TAK patients was significantly higher than that of the healthy control group. In CD3^+^IFN-γ^+^ cells, the proportion of CD3^+^CD8^+^IFN-γ^+^cells was significantly increased in TAK patients. In conclusion, our results indicated CD8^+^ T lymphocyte is another main source of IFN-γ production in TAK.

Consistent with our results, lots of studies demonstrated the important role of CD8^+^ T cells in autoimmune diseases. In a giant cell arteritis case of a woman at the age of 31, predominant CD8^+^ T lymphocyte and multinucleated giant cells infiltrations were important characteristics in the pathological findings^11^. Samson et al. found that polyclonal stimulation of CD8^+^ T cells was associated with the severe of vasculitis^12^. In polyarteritis nodosa (PAN) patients, rather than the number of CD4^+^ T cells infiltrating, that of CD8^+^ T cells in the arteries intima was significantly higher. This phenomenon explained the effect of CD8^+^ cytotoxic T lymphocytes (CTL) in the development of vascular injury in PAN^13^. In a systemic lupus genotype study, CD8^+^ T cells was proved to be the only one strong prediction of remaining relapse-free^14^. In murine model of Kawasaki Disease, either the excessive activation or the imbalance between activation and inhibition of CD8^+^T cells contributed to progress of coronary arteritis^15^. In some cases, the percentage of CD8^+^HLA-DR^+^T cells was considered an optimal index in diagnosing Kawasaki Disease^16^. Some studies also found that the CD8^+^ lymphocyte infiltration and the CD4^+^/CD8^+^lymphocyte ratio could distinguish GCA from TAK well^17–19^. Instead of CD4^+^ T cell values, significantly higher CD8^+^ T cell was found in TAK patients when comparing to controls^20^.

CD8^+^ T cells infiltrating the arterial wall produced cytotoxic molecules (granzymes and perforin) and cytokines (IL-17 and IFN-γ) ^21^. This may partly explain the reason why the CD3^+^CD8^+^IFN-γ^+^/CD3^+^IFN-γ^+^ ratio was positively correlated with serum IL-17 level in our study. CD8^+^ T cells played very important role in upstream of vascular remodeling pathways. The cytotoxic molecules and IFN-γ produced by CD8^+^ T cells triggered the recruitment of monocytes ^21^. IFN-γ affects IL-12R expression, cytotoxic T lymphocyte (CTL) proliferation and immune dominance in CD8^+^ T cells via the IFN-γ-R^22^. In vivo, IFN-γ produced by CTL induced skin graft rejection and decelerated CTL motility in tissue. In vitro, IFN-γ was mainly responsible for the motility and speed of CD8^+^ T cells. Antigen-specific contact-mediated T-cell killing was also promoted by IFN-γ. CTL cytolytic function in inflammation was enhanced by the environment rich in IFN-γ, CD4^+^T helper 1 cells and natural killer T cells and paracrine and autocrine mechanisms of signaling ^7^.

IFN-γ stimulated monocytes differentiating into macrophages when the monocytes recruited in the arterial. IL-1β, IL-6 and TNF-α produced by macrophages amplify the inflammatory response and lead to the large vessel vasculitis which including fever, weakness, anorexia, weight loss and acute phase response^23^. While the IFN-γ-activated macrophages released mediators, which were toxic to the arterial tissue. Besides, matrix metalloproteinase-2 (MMP-2), MMP-9, nitric oxide (NO) and reactive oxygen species caused destruction of the media and digestion of the internal elastic lamina, which could promote vascular wall remodeling ^21^.

The main limitations of this study were the retrospective design and its relatively small sample size. Another limitation is the CD3^+^CD8^+^IFN-γ^+^cells phenotypes were only analyzed in peripheral blood specimens. Therefore, further studies are required to better understand the role of CD3^+^CD8^+^IFN-γ^+^cells in TAK patients. Above all, we found that the proportion of CD3^+^CD8^+^IFN-γ^+^cells in CD3^+^IFN-γ^+^ cells in TAK patients were significantly increased and positively correlated with serum IFN-γ level. The increasing IFN-γ secreted by CD8^+^ T cells in peripheral blood of TAK patients is an important mechanism involved in the injury of TAK vessel wall.

## Supplementary Information

Supplementary Legend.
