# Supplementary material for: CD8+ T lymphocyte is a main source of interferon-gamma production in Takayasu’s arteritis
Source: Sci Rep. 2021 Aug 24;11:17111. doi: 10.1038/s41598-021-96632-w (PMC8384861; doi:10.1038/s41598-021-96632-w)
Supplement: Supplementary file 1 — Supplementary Legend. [file 41598_2021_96632_MOESM1_ESM.docx]

**CD8^+^ T lymphocyte is a main source of interferon- gamma production in Takayasu’s arteritis**

Yan-Long Ren^1, #^, Tao-Tao Li^2, #^, Wei Cui^3^, Li-Min Zhao^3^, Na Gao^2^, Hua Liao^2^, Jiang-Hui Zhang^3^, Jun-Ming Zhu^4^, Zhi-Yu Qiao^4^, Shi-Chao Guo^4^, Li-Li Pan ^2*^

**^1^Yan-Long Ren,** MM, Department of Cardiology, Capital Medical University Affiliated Beijing Anzhen Hospital, Beijing Lab for Cardiovascular Precision Medicine, Beijing, China

**^2^Tao-Tao Li,** MD, Department of Rheumatology, Capital Medical University Affiliated Anzhen Hospital, Beijing, China

**^3^Wei Cui,** MB, Department of Rheumatology, Capital Medical University Affiliated Anzhen Hospital & Beijing Institute of Heart, Lung and Vessel disease, Beijing, China

**^3^Li-Min Zhao,** MB, Department of Rheumatology, Capital Medical University Affiliated Anzhen Hospital & Beijing Institute of Heart, Lung and Vessel disease, Beijing, China

**^2^Na Gao,** MD, Department of Rheumatology, Capital Medical University Affiliated Anzhen Hospital, Beijing, China

**^2^Hua Liao,** MM, Department of Rheumatology, Capital Medical University Affiliated Anzhen Hospital, Beijing, China

**^3^Jiang-Hui Zhang**, MD, Department of Rheumatology, Capital Medical University Affiliated Anzhen Hospital & Beijing Institute of Heart, Lung and Vessel disease, Beijing, China

^4^**Jun-Ming Zhu**, MD, Department of cardiovascular surgery, Capital Medical University Affiliated Anzhen Hospital, Beijing, China

^4^**Zhi-Yu Qiao**, MM, Department of cardiovascular surgery, Capital Medical University Affiliated Anzhen Hospital, Beijing, China

^4^**Shi-Chao Guo**, MM, Department of cardiovascular surgery, Capital Medical University Affiliated Anzhen Hospital, Beijing, China

**^2^Li-Li Pan,** MD, Department of Rheumatology, Capital Medical University Affiliated Anzhen Hospital, Beijing, China

**^#^** Yan-Long Ren and Tao-Tao Li contributed equally to this article.

*** Corresponding author：**Li-li Pan, Department of Rheumatology, Capital Medical University Affiliated Anzhen Hospital, 2 Anzhen Road, Chaoyang District, Beijing, China, E-mail: [lilypansxmu@sina.com](mailto:lilypansxmu@sina.com). Tel: +86-10-64456252, Fax: +86-10-64456252.

**Running title:** Interferon-γ-producing CD8^+^ T cells in Takayasu’s arteritis


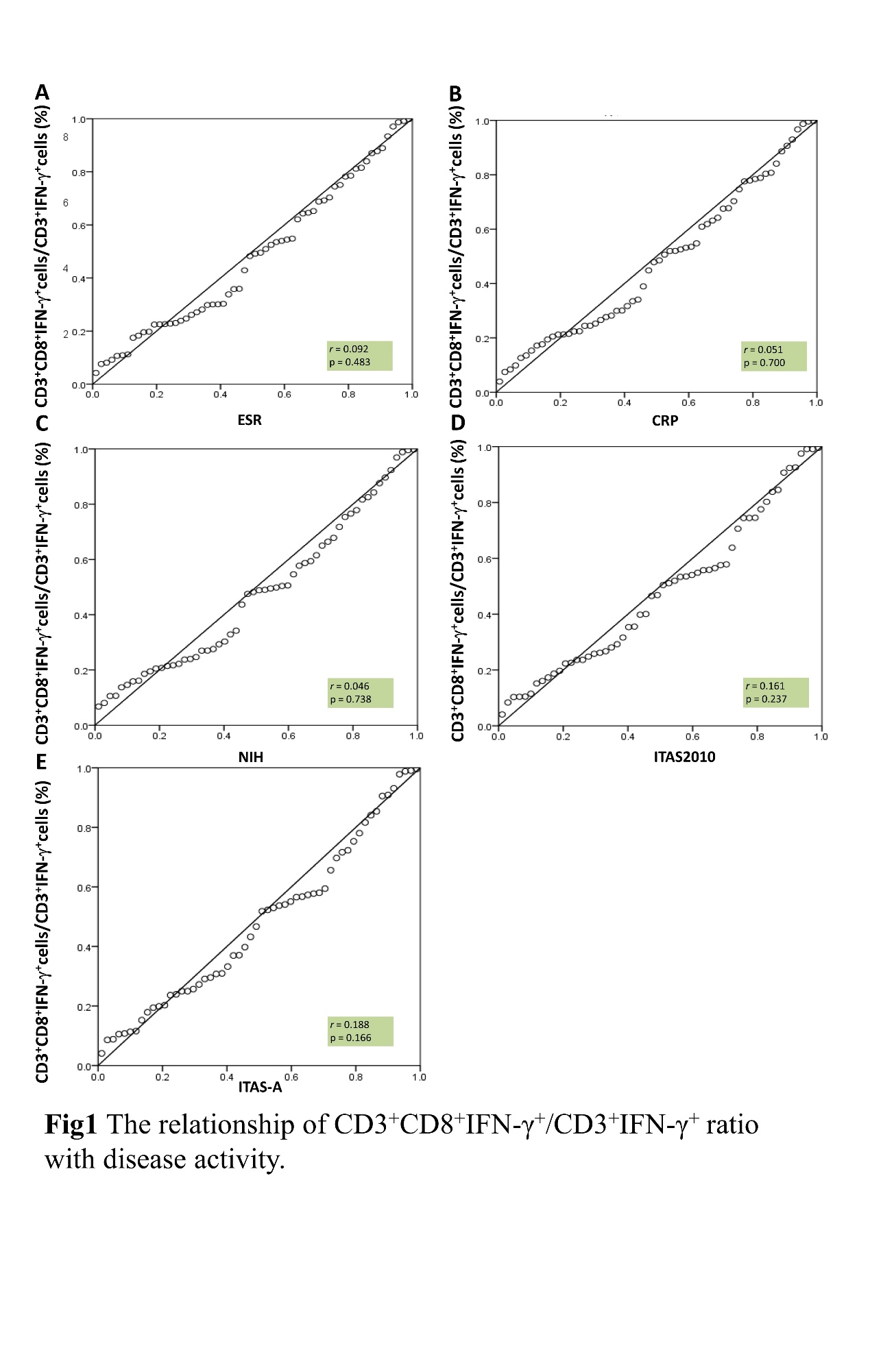


**Supplementary Fig 1.** The relationship of CD3^+^CD8^+^IFN-γ^+^/CD3^+^IFN-γ^+^ ratio with disease activity. The CD3^+^CD8^+^IFN-γ^+^/CD3^+^IFN-γ^+^ ratio was negatively correlated with (A) ESR (r=0.092, p=0.483), (B) CRP (r=0.051, p=0.700) , (C) NIH(r=0.046, p=0.738), (D) ITAS2010 (r=0.161, p=0.237) and (E) ITAS-A (r=0.188, p=0.166).
